# The Incidence of Posttraumatic Stress Symptoms in Children

**DOI:** 10.5435/JAAOSGlobal-D-22-00245

**Published:** 2023-08-11

**Authors:** Collin May, Patricia E. Miller, Manahill Naqvi, Emily Rademacher, Justyna Klajn, Daniel Hedequist, Benjamin J. Shore

**Affiliations:** From the Department of Orthopedic Surgery, Boston Children's Hospital, Boston, MA.

## Abstract

**Objective:**

To determine the incidence of posttraumatic stress disorder (PTSD) symptoms and risk factors for their development in children and adolescents undergoing orthopaedic surgery for trauma.

**Design:**

Prospective cohort study.

**Setting:**

Level-1 trauma center.

**Patients:**

Children (8 to 18), undergoing surgery for orthopaedic trauma.

**Intervention:**

Upper and lower extremity surgery for orthopaedic trauma.

**Main outcome measurements:**

PTSD symptoms at postoperative follow-up as determined by the Child PTSD Symptom Scale (CPSS).

**Results:**

A total of 176 children with an average age at surgery of 13 years (8 to 18.8 years) participated in the study. Twenty-six subjects had high levels of PTSD symptoms (15%; 95% CI = 10.0 to 21.1%). Univariable and multivariable analyses determined that female sex (OR 2.63, 95% CI = 1.06 to 6.67, *P* = 0.04), older age at surgery (OR 1.25, 95% CI = 1.04 to 1.51, *P* = 0.02), and undergoing a previous surgery (OR 2.86, 95% CI = 1.06 to 7.73, *P* = 0.04) were all associated with increased PTSD risk.

**Conclusions:**

Children and adolescents experience a high level of PTSD symptoms (15%) after surgery for orthopaedic trauma. Clinicians should be aware of PTSD symptoms in children and adolescents after surgery for orthopaedic injuries and use comprehensive screening to facilitate timely intervention and treatment.

**Level of Evidence:**

II.

Annually, in the United States, more than 20 million children are injured, resulting in more than eight million visits to emergency facilities, 10 million primary care visits, and 200,000 admissions to the hospital.^1-3^ Injuries continue to represent the number one cause of death for children aged 1 to 18 and result in tremendous morbidity and permanent disability.^4^

In addition to the musculoskeletal trauma of injury, children suffer additional psychological sequela secondary to their events that can be life-altering, with lasting implications on relationships, activities, school, and the process of recovery. It is clear that children suffering from traumatic injuries can also be suffering from posttraumatic stress disorder (PTSD); which if left untreated, can be associated with negative functional outcomes, decreased quality of life, cognitive impairments, dysfunctional social and family relationships, and increased utilization of health services.^5,9^ These negative effects associated with PTSD can extend into adult life with a greater risk of several mental and physical impairments of adulthood, including cardiovascular, endocrine, digestive, and musculoskeletal diseases; chronic health conditions; substance abuse; and depression.

It is common for both children and parents to experience some degree of severe traumatic stress reaction in the first month after injury,^7,10^ and studies of the prevalence of posttraumatic stress (PTS) in this population suggest a rate of 13% to 45%.^11,14^ Stress reactions after trauma can serve an adaptive purpose because psychological recovery may necessitate some re-experiencing of the event, balanced by avoidance of distressing reminders.^15^ Stress reactions have the potential to be disruptive, affecting activities of daily living and hinder the process of recovery. Function impairment associated with a combination of PTS symptoms can result in the diagnosis of acute stress disorder or PTSD.^16^ A recent meta-analysis of 18 studies looking at posttraumatic stress after illness and injury found that approximately 20% of injured children will experience posttraumatic symptoms.^17^ These symptoms have been documented after various types of pediatric injury, including burns,^6^ road traffic accidents,^7,10,18^ interpersonal violence,^19^ and both major and minor orthopaedic injury.^20,21^ To diagnose PTSD in adults and children over 6 years, there must be at least one symptom of intrusion, two symptoms of avoidance or negative alteration in cognition or mood, and two symptoms of arousal present.

The purpose of this study was to report the incidence of PTSD symptoms in children and adolescents who were treated surgically for orthopaedic trauma and identify risk factors associated with the development of PTSD symptoms in this cohort. We hypothesized that PTSD symptoms after orthopaedic surgery would be more commonly present in those children who had previously undergone surgery.

## Methods

We performed a prospective observational study on a convenience sample of children aged 8 to 18 years, inclusive, undergoing surgery between June 14th, 2014, and April 27th, 2016, for orthopaedic trauma at a single level-1 trauma center (16 pediatric orthopaedic surgeons on the call roster) in the Northeast United States. The study was institutional review board approved, and consent/assent was obtained from each parent/legal guardian and/or child. All patients were initially evaluated and consented at least 30 days from the date of surgery.

The inclusion criteria were patients sustaining musculoskeletal trauma for which orthopaedic surgery was performed. The exclusion criteria were patients with preexisting psychologic disorders (determined by self-report or from review of the medical record), those with traumatic brain injury, and those whose primary language was not English. Patients were identified for study participation based on the review of surgical and clinic schedules by a member of the research team not involved in clinical care, who identified and approached patients and families during their postoperative clinical visit.

Patient, injury, injury severity score, and treatment characteristics were recorded for the entire cohort. Assessment of PTSD symptoms occurred at initial presentation using the Child PTSD Symptom Scale (CPSS), a validated self-report indicator of PTSD symptoms completed by children aged 8 to 18 years of age.^20-22^ The CPSS represents a pediatric variety of the Posttraumatic Diagnostic Scale (PTDS), which in adults is an accepted measure of PTSD severity and diagnosis.^22^ The CPSS was adapted to included developmentally appropriate language to maximize pediatric comprehension. The CPSS has shown good reliability and was compared with the Child Posttraumatic Stress Reaction Index (CPTSD-RI), an extensively validated measure for PTSD symptoms in children after trauma.^22^ The CPSS contains 17 questions regarding PTSD symptoms scored on a 4-point Likert scale (0 = not at all, only at one time; 1 = once per week or less, once in a while; 2 = 2 to 4 times per week, half the time; 3 = 5 or more times per week, almost always). A cutoff score of 11 on the CPSS previously produced sensitivity and specificity of 95% and 96%, respectively, for symptoms consistent with a PTSD diagnosis.^13^

## Statistical Analysis

Binary and categorical data were summarized by frequency and percent, whereas continuous data were summarized by mean and standard deviation (SD), median and range, or median and interquartile range (IQR, 25th to 75th percentile) when data deviated from normality. The incidence of PTSD risk was estimated by the proportion of subjects who scored at least 11 points on the CPSS scale. A 95% confidence interval (CI) was calculated. Univariable and multivariable logistic regression were used to determine the potential risk factors for PTSD risk. Risk factors analyzed included age at surgery, sex, race, ethnicity, household income, fracture location, mechanism of injury, injury severity score, length of hospital stay, previous surgery, and previous hospitalization. A step-wise model selection procedure was used based on Akaike information criterion to obtain the best-fitting multivariable model for PTSD risk. Odds ratios with corresponding 95% confidence intervals were estimated for all significant factors. All tests were two-sided, and *P* < 0.05 were considered significant.

Power analysis, conducted a priori, indicated that to estimate 95% confidence interval with a width of 0.1 around an estimated incidence of PTSD risk of 19%, we would have required a sample of at least 236 subjects. To meet this estimate, we initially enrolled 250 subjects; however, we ended up with complete data on only 176 with an incidence rate of 14.8%. A post hoc power analysis was conducted and determined that we were able to estimate a two-sided 95% confidence interval with a width of 0.105 around an estimate of 0.148.

Statistical analysis was performed using R software, version 3.0.0 (Vienna, Austria).

This study was funded by the Pediatric Orthopedic Society of North America with a Start Up Grant (2015) $10,000.

## Results

**Figure 1:**
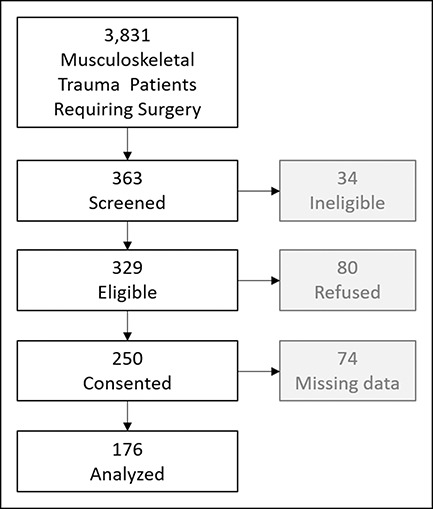
Strobe diagram of patients eligible, screened. and participated in the current study.

**Figure 2:**
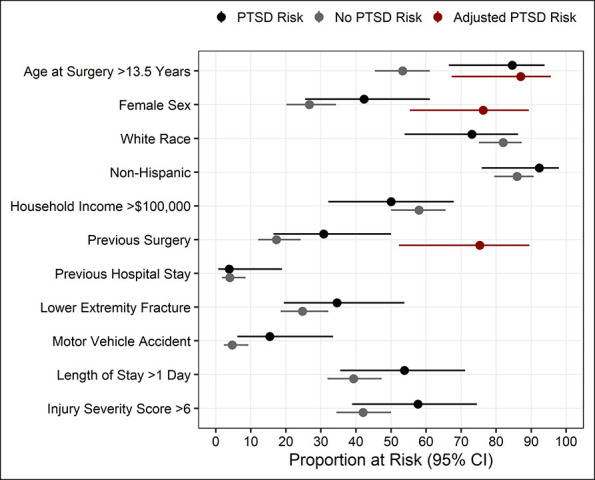
Forest plot of patient characteristics by posttraumatic stress disorder risk.

During the study period a total of 3,831 trauma surgeries were performed, from which a convenience sample of 363 subjects were approached for study participation. Of these 363 subjects, 34 were ineligible on further questioning, 79 refused to participate, and 250 were consented and enrolled. Of these 250 subjects, 176 completed a CPSS questionnaire between 1 and 6 months postsurgically and were included in the analysis; the remaining 74 did not return for completion of the CPSS questionnaire (Figure 1). This cohort of 176 patients was assessed for PTSD symptoms at a median of 66 days from surgery, had an average age at surgery of 13 years (range 8.0-18.8 years), and was 71% male children (Table 1). Twenty-six (14.7%; 95% CI = 10.0 to 21.1%) subjects scored a high level of PTSD symptoms, with CPSS scores of 11 or higher (median, 17; range, 11 to 42). In contrast, those who scored below 11 had a median CPSS score of 3 (range, 0 to 10). A multivariable analysis determined that patient sex, age at surgery, and undergoing a previous surgery were each associated with PTSD symptom risk (Figure 2, Table 2). Female children demonstrated an increased odds of being at risk for PTSD symptoms (OR 2.63, 95% CI = 1.06 to 6.67, *P* = 0.04). For each additional year of age at surgery, the odds of being at risk for PTSD symptoms increased by 25% (OR = 1.25; 95% CI = 1.04 to 1.51; *P* = 0.02). Subjects who reported having a previous surgery had nearly 3 times the odds of being at risk for PTSD symptoms (OR = 2.86; 95% CI = 1.06 to 7.73; *P* = 0.04).

**Table 1:** Patient Cohort N = 176.

Variable	Freq.	(%)
Age at surgery (*years; mean ± SD*)	13.4	± 2.68
Sex (*% male*)	125	71%
Time of CPSS administration from surgery (days)	66	30-182
Race		
Caucasian	142	81%
Black or African American	15	9%
Asian	5	3%
Other/unknown	14	8%
Ethnicity		
Hispanic or Latino	13	7%
Not Hispanic or Latino	153	87%
Other/unknown	10	6%
Household income^a^		
<$10,000	4	2%
$10,000-39,999	15	9%
$40,000-69,999	20	11%
$70,000-99,999	18	10%
>$100,000	100	57%
Previous surgery	34	19%
Previous hospitalization	7	4%
Fracture location		
Upper extremity	99	56%
Lower extremity	78	44%
Hip, spine, or skull	1	1%
Visceral	1	1%
Method of injury^a^		
Motor vehicle accident	11	6%
Sports	95	54%
Playing	52	30%
Other/Unknown	18	10%
Injury severity score (*median[IQR]*)	4	4-8
Length of stay		
More than a day	46	26%
Length of stay of those over one day (*median[IQR]*)	4	3-4

CPSS = Child PTSD Symptom Scale, IQR = interquartile range

a Household income data only available for 142 subjects, and the method of injury data only available for 146 subjects.

**Table 2:** Comparisons Across PTSD Risk

Characteristic	At risk for PTSD (n = 26)	No evidence of risk (n = 150)	Freq.	(%)	P	Adj P^a^	OR (95% CI)
	Freq.	(%)					
Age at surgery (*years; mean ± SD*)	14.4	± 2.25	13.2	± 2.72	0.04	0.02	1.25 (1.04-1.51)
Sex (*% female*)	11	42%	40	27%	0.11	0.04	2.63 (1.06-6.67)
Race							
Caucasian	19	73%	123	82%	—		
Black or African American	3	12%	12	8%	0.49		
Asian	2	8%	3	2%	0.12		
Other/unknown	2	8%	12	8%	0.93		
Ethnicity							
Hispanic or Latino	0	0%	13	9%	0.99		
Not Hispanic or Latino	24	92%	129	86%			
Other/unknown	2	8%	8	5%			
Household income							
<$10,000	1	4%	3	2%	0.20		
$10,000-39,999	4	15%	11	7%			
$40,000-69,999	2	8%	18	12%			
$70,000-99,999	2	8%	16	11%			
>$100,000	13	50%	87	58%			
Previous surgery	8	31%	26	17%	0.12	0.04	2.86 (1.06-7.73)
Previous hospitalization	1	4%	6	4%	0.97		
Fracture location							
Upper extremity	10	39%	89	59%	0.22		
Lower extremity	15	58%	63	42%	0.14		
Method of injury							
Motor vehicle accident	4	15%	7	5%	—		
Sports	11	42%	84	56%	0.04		
Play	9	35%	43	29%	0.17		
Injury severity score (*median[IQR]*)	8	4-8	4	4-8	0.12		
Length of stay	9	35%	37	25%	0.29		
More than one day	3	3-4	4	3-4	0.86		

IQR = interquartile range, PTSD = posttraumatic stress disorder

a Adj P, adjusted *P*-values based on multivariable analysis. Variables without *P*-values were removed from the multivariable model based on model fit statistics.

## Discussion

The primary goal of this study was to calculate the incidence of and risk factors for the development of PTSD symptoms in a pediatric population treated surgically for orthopaedic trauma. We found that nearly 15% of all patients enduring surgery for orthopaedic trauma experienced a high level of PTSD symptoms. This observation was consistent even after relatively minor injuries, such as common forearm and elbow fractures. Aaron et al^11^ found a similar rate of PTSD in children at 1 month after injury, reporting an incidence of 23%. In contrast, two recent studies in pediatric orthopaedic populations demonstrated a moderately higher rate of PTSD development 1 to 3 months after orthopaedic injuries of approximately 33%.^20,21^ Our lower incidence of PTSD symptoms may be explained by a longer period of survey acquisition (1-6 months), where symptoms which were present in the first 1 to 3 months could have subsided.

An analysis of risk factors associated with the occurrence of PTSD symptoms demonstrated several notable findings. Interestingly, we did not appreciate relationships between injury severity and the development of PTSD symptoms. Although one might hypothesize that the physical trauma of more serious injury may correlate with an increased likelihood of emotional difficulty during recovery, this association was not seen. A lack of connection between injury severity and development of PTSD is consistent with previous studies.^20,23-25^ In this study, age and female sex were both found to be independent predictors for the development of PTSD. Several meta-analyses have identified increasing age as a risk factors for PTSD development after a range of traumatic events including after earthquakes,^26^ and general trauma.^17,27^ Sex differences in the development of PTSD have been previously reported in adolescents, with women at higher risk for the development of PTSD.^27,28^ In our study, we found sex to be an independent risk factor, with female sex demonstrating a 2.63 times greater odds of developing PTSD symptoms.

A history of previous trauma has been found to be a risk factor associated with the development of PTSD after injury. Daviss et al^12^ found a strong association between exposure to previous traumatization and the development of future PTSD in their population of injured children. Similar association between previous traumatization and development of PTSD has been found with noninjury trauma.^29^ In our cohort, a history of previous surgery (which we would consider a traumatic event in the life of a child) was associated a 3 times greater odds of developing PTSD symptoms.

This study is not without limitations. Patients for this study were recruited using convenience sampling, which could have introduced sampling bias to the results and can limit generalizability. Because the sampling was based on research staff availability at our outpatient clinics, we do not feel this was likely to bias PTSD incidence one way or another. There was an increased number of male children in our cohort, which could relate to sampling bias or could be related to the fact that male children may be participating more frequently in activities where they suffer injuries which require orthopaedic surgery. We evaluated for PTSD symptoms at a single, early time-point in the clinical course postinjury. It is possible that a child's PTSD symptoms may increase or decrease after 6 months from the initial injury. Continued follow-up of this cohort is planned in part to address this potential limitation. Another limitation is the lack of a control group with musculoskeletal injury who did not receive surgical intervention, thus we are not able to distinguish the relative effect of the surgery apart from the musculoskeletal injury alone. Unfortunately, we did not capture items related to family dynamics or family dysfunction in this cohort, and it is possible that family dysfunction could have influenced the prevalence of PTSD in our sample. In future prospective study, we will gather information regarding family dynamics to understand the interplay between that and the development of PTSD. In addition, we excluded patients with a history of psychologic disorders, a group that may be at increased risk for development of PTSD symptoms. This may have falsely reduced the incidence of PTSD in our cohort and reduces the generalizability of the results. The high rate of PTSD symptoms after surgery for MSK injury may therefore be even higher than that reported, further supporting the need for more comprehensive screening. Finally, in reviewing the demographics of our cohort, we acknowledge that the mean household income was higher than the US median and represents the patient population surrounding our hospital; this may reduce the generalizability of the results at other large urban pediatric hospitals within the United States.

**Figure 3:**
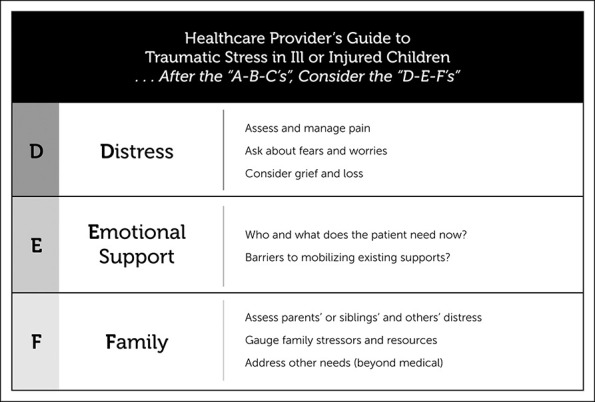
D-E-F Pocket cards from the Trauma Stress Toolkit for Healthcare Providers.

*Despite these limitations, we found that the prevalence of PTSD symptoms in a convenience sample of children and adolescents who were treated surgically for orthopaedic trauma was 15%.* We feel important *recommendations* regarding the incidence of posttraumatic stress symptoms after orthopaedic trauma surgery can be made. *The high rate of PTSD symptoms highlights the need for comprehensive screening of all children and adolescents at risk, regardless of family income, such that appropriate intervention and referral may take place.* There is an extensive body of literature regarding effective treatments of pediatric PTSD, and many recent efforts have been focused on secondary prevention of persistent symptoms through early intervention.^30^ Universal preventive strategies have been successfully used in many institutions, and the ‘D-E-F’ protocol of the National Child Traumatic Stress Network is an approach that applies guidelines of trauma-informed care in an easy-to-remember and effective manner (Figure 3).^31^ Accurate identification of individuals at higher risk for the development of PTSD is of paramount importance to target treatments and ensure proper referral to mental health providers, with the goal of limiting the potential late mental and physical sequelae of PTSD.
